# Sex Differences in Sex Hormone Profiles and Prediction of Consciousness Recovery After Severe Traumatic Brain Injury

**DOI:** 10.3389/fendo.2019.00261

**Published:** 2019-04-26

**Authors:** Yu H. Zhong, Hong Y. Wu, Ren H. He, Bi E. Zheng, Jian Z. Fan

**Affiliations:** Department of Rehabilitation Medicine, Nanfang Hospital, Southern Medical University, Guangzhou, China

**Keywords:** traumatic brain injury, sex hormones, consciousness, sex, differences

## Abstract

**Objective:** The clinical course of unconsciousness after traumatic brain injury (TBI) is commonly unpredictable and it remains a challenge with limited therapeutic options. The aim of this study was to evaluate the early changes in serum sex hormone levels after severe TBI (sTBI) and the use of these hormones to predict recovery from unconsciousness with regard to sex.

**Methods:** We performed a retrospective study including patients with sTBI. A statistical of analysis of serum sex hormone levels and recovery of consciousness at 6 months was made to identify the effective prognostic indicators.

**Results:** Fifty-five male patients gained recovery of consciousness, and 37 did not. Of the female patients, 22 out of 32 patients regained consciousness. Male patients (*n* = 92) with sTBI, compared with healthy subjects (*n* = 60), had significantly lower levels of follicular stimulating hormone (FSH), testosterone and progesterone and higher levels of prolactin. Female patients (*n* = 32) with sTBI, compared with controls (*n* = 60), had significantly lower levels of estradiol, progesterone, and testosterone and significantly higher levels of FSH and prolactin. Testosterone significantly predicted consciousness recovery in male patients. Normal or elevated testosterone levels in the serum were associated with a reduced risk of the unconscious state in male patients with sTBI. For women patients with sTBI, sex hormone levels did not contribute to the prediction of consciousness recovery.

**Conclusion:** These findings indicate that TBI differentially affects the levels of sex-steroid hormones in men and women patients. Plasma levels of testosterone could be a good candidate blood marker to predict recovery from unconsciousness after sTBI for male patients.

## Introduction

Traumatic brain injury (TBI) is a major cause of death and disability worldwide and is increasing in incidence (1). Patients with acute severe TBI (sTBI) often develop severe disorders of consciousness, i.e., coma, minimally conscious state or vegetative state. Although many patients may regain consciousness during the 1-month post-TBI period, the minimal conscious state may also develop into a chronic and even permanent state (2). Early detection of consciousness in patients with TBI could predict subsequent recovery of neurological function since early recovery of consciousness is closely related to better long-term functional outcomes (3). However, there is an ongoing debate about the clinical assessment of consciousness, which relies on inferences obtained from observed responses to external stimuli. This clinical evaluation of consciousness may be erroneous in 40% of patients, since the responses of patients with severe brain damage may be very limited (4, 5). In addition, rehabilitative care will be limitedly accessible to those who are inaccurately identified as poor prognoses due to the lack of a tool for predicting consciousness recovery (6). Hence, it is crucial to find a biomarker to predict the recovery of consciousness for patients suffering from TBI.

Hormone dysfunction, also known as post-TBI hormonal deficiency syndrome, is very common in the post-acute phase of sTBI. It has been reported that up to 80% of patients with sTBI suffer from some types of acute hypopituitarism and related hypogonadism (7, 8). The literature suggests that sex hormones can affect damage after TBI and are associated with the stress response occurring in the acute phase of the disease. Furthermore, there is proof that estrogen and progesterone have neuro-protective effects, suggesting that inadequate levels may have both acute and long-term consequences on the recovering brain (9). Decades of studies show that testosterone levels are low in 36.5–100% of patients with sTBI, however, the prognostic significance of testosterone levels remains controversial (10). Although insufficiency in hormones after TBI has become increasingly recognized, there are limited data focusing on TBI survivors regarding the role of sex hormones in predicting consciousness.

There is increasing evidence demonstrating significant sex differences in the nervous system response to traumatic injury (11). A growing number of studies in experimental TBI report that female brains consistently exhibit less damage in comparison to their male counterparts because of effects of gonadal steroid hormones at time of injury (8). However, studies regarding the influence of sex on outcomes and recovery of TBI are still scarce. To the best of our knowledge, there is no previous study investigating the association between serum hormone levels during the acute TBI phase and the recovery of consciousness in patients with TBI. The goals of this study were to assess sex differences in alterations of serum sex hormones after sTBI and determine whether sex hormones can effectively predict recovery of consciousness with regard to sex.

## Methods

### Patients and Definitions

We retrospectively screened all patients with TBI admitted to the neurosurgery, emergency or rehabilitation department of our institution from 2007 to 2017. The inclusion criteria were as follows: (1) age of 18–75 years old; (2) head trauma with Glasgow Coma Scale (GCS) score of 3–8 based on the first score registered after resuscitation, with no eye opening for at least 24 h; (3) absence of previous neurologic disorders; (4) absence of a previous history of breast cancer requiring chemotherapy treatment/tamoxifen, pituitary, or hypothalamic tumor, prostate cancer receiving orchiectomy, or hormone suppression agents, or untreated thyroid disease; (5) serum sex hormone measurement received within 1 week after trauma. Ninety-two male patients and 32 female patients with sTBI were enrolled in this study following above-mentioned criteria. Healthy subjects were separately enrolled as controls for serum sex hormone measurement. Healthy subjects had no history of neurological, psychiatric, cardiovascular, pulmonary, renal or endocrinological disease, and had not received replacement hormone therapy or contraception. In addition, control women were interviewed about their menopausal status and reproductive history. If this information was not available, subjects >50 years of age were defined as post-menopausal. Sixty age-and sex-matched healthy controls were included for both male and female group.

All patients were given both oral and written information about the study and a written informed consent was obtained.

### Parameters

A standardized case collection form was used to determine the causes of trauma, age, sex, injury severity score (ISS), GCS scores, and neuroradiological data at baseline. The severity of the trauma was evaluated by ISS. The lowest recorded GCS scores before sedation and intubation from the emergency department or scene of accident was used in this study. The type of injury was obtained from initial head computed tomography (CT) report.

Serum sex hormone measurements for all patients were performed in 1 week after sTBI. Additionally, serum samples for premenopausal females were collected either in the follicular phase (days 5–10) or the luteal phase (days 18–23) of their cycle. Blood for enrolled patients was primarily collected in the morning (~7:00 a.m.) for analysis of estradiol, follicular stimulating hormone (FSH), luteinizing hormone (LH), progesterone, prolactin, and testosterone. All sex hormones were analyzed at the accredited clinical chemistry laboratory at Nanfang Hospital, Southern Medical University. Serum estradiol, progesterone, and testosterone were analyzed using radioimmunoassay with the Coat-A-Count *in-vitro* diagnostic test kit (Siemens Healthcare Diagnostics Inc., Los Angeles CA). Serum FSH, LH, and prolactin were measured by electrochemiluminescence immunoassay (ECLIA; Modular Analytics E170, Roche, GmbH, Hannheim, Germany). Male patients were divided into two subgroups according to the normal range (1.80–8.82 ng/ml) of male testosterone provided by the accredited clinical chemistry laboratory. Testosterone levels <1.80 ng/ml was classified as low testosterone level group, and testosterone levels >1.80 was classified as normal or elevated testosterone level group.

### Study Outcome

The primary outcome was consciousness recovery. All enrolled patients were classified into two groups according to their final coma recovery result: recovery of consciousness (RC) and no recovery of consciousness (NRC). Patients were considered to be the RC group if they met at least one of the following demonstrations: (1) functional use of one or more objects, (2) functional interactive communication, or (3) clearly discernable behavioral manifestation of a sense of self. The judgment on the unconscious state during the follow-up period was evaluated by the Coma Recovery Scale–Revised (CRS-R) (12). The patients were followed for at least 6 months.

### Statistical Analysis

Normally distributed data are presented as the mean ± standard deviation (SD) and compared using Student's *t* test. Non-normally distributed continuous data are presented as median (interquartile range) and compared by the Mann- Whitney U test. Chi-square or Fisher's exact tests were performed to compare categorical data. Independent variables were screened to select those with statistically significant differences between the RC and NRC groups using single-factor analysis. Logistic regression analysis was used to determine which variables independently predicted recovery of consciousness. A logistic regression model contained sex hormones and clinical predictors including age, pupil reactivity, GCS score, ISS, and computed tomography (CT) characteristics (Rotterdam CT classification). The times to recovery of consciousness for patients with sTBI were illustrated with Kaplan–Meier curves and compared using the Cox proportional hazards regression model in hazard ratios (HR), with adjustment for baseline characteristics. The prediction of recovery of consciousness was analyzed using the receiver operating characteristic (ROC) curve method. A *p*-value of < 0.05 was considered statistically significant. All analyses were two-sided and performed using SPSS software version 21.0 (SPSS Inc., Chicago, IL, USA).

## Results

### Enrollment and Characteristics of the Patients

Of 3,411 patients with TBI screened for eligibility, 124 patients with sTBI met all inclusion criteria and were enrolled in this study. Thirty-two of 124 patients were women. The primary mechanism of injury was motor vehicle collisions in both men and women. The median GCS at admission for men and women was 4 and 5, respectively. The median ISS score was 36 in both men and women. No sex differences were found in the types of injury observed by head CT, demographic, and injury variables including age, GCS score at admission, and ISS. The baseline characteristics of the patients are shown in Table 1.

**Table 1:** Characteristics of patients at baseline.

**Characteristic**	**Men (*n* = 92)**	**Women (*n* = 32)**	***P*-value**
**AGE (YEARS)**			
Median (IQR)	35 (23–45)	42 (27–48)	0.278
**CAUSE OF INJURY (NO.%)**			
Automobile/motorcycle	57/92 (61.96%)	16/32 (50.00%)	0.236
Fall/jump	16/92 (17.39)	9/32 (28.13%)	0.192
Other	19/92 (20.65%)	7/32 (21.88%)	0.884
**RADIOLOGICAL INJURY TYPE**			
Subdural hematoma	37/92 (40.22%)	11/32 (34.38%)	0.559
Diffuse axonal injury	18/92 (19.57%)	6/32 (18.75%)	0.920
Epidural hematoma	14/92 (15.22%)	3/32 (9.38%)	0.556
Subarachnoid hemorrhage	24/92 (26.09%)	10/32 (31.25%)	0.573
Contusion	49/92 (53.26%)	21/32 (65.63%)	0.224
Intraventricular hemorrhage	6/92 (6.52%)	2/32 (6.25%)	0.957
Intracerebral hemorrhage	13/92 (13.04%)	9/32 (28.13%)	0.074
GCS at admission, median	4 (3–6)	5 (3–6)	0.652
ISS, median	36 (30–42)	36 (28–42)	0.514
**DAYS FROM INJURY**			
To measure the level of sex hormone (days)	4.62 ± 1.77	5.09 ± 2.18	0.235

*GCS, Glasgow Coma Scale; ISS, Injury Severity Score; IQR, interquartile range. Results are given as median (IQR) or n (%). Overall scores on the GCS range from 3 to 15, with lower scores indicating a lower level of consciousness. The ISS ranges from 0 to 75, with higher scores indicating greater severity of injury*.

### Serum Sex Hormone Levels by Sex After sTBI

Table 2 summarizes serum sex hormones by sex for estradiol, FSH, LH, progesterone, prolactin, and testosterone for patients and healthy controls. Serum estradiol levels were significantly lower in women with sTBI than observed in matched healthy subjects (58.50 ± 43.79 vs. 94.69 ± 73.66 pg /ml; *p* = 0.013), whereas levels were similar in control values for men. The mean FSH levels for men with sTBI were lower than those for their controls (3.56 ± 3.50 vs. 5.27 ± 2.89 mIU/L; *p* = 0.002). In contrast, FSH levels in women with sTBI were higher than those in their controls (17.76 ± 12.78 vs. 8.87 ± 5.93 mIU/L; *p* < 0.001). Mean prolactin levels for both men (26.91 ± 14.35 vs. 10.00 ± 4.69 ng/ml; *p* < 0.001) and women (52.77 ± 23.26 vs. 18.89 ± 10.26 ng/ml; *p* < 0.001) were significantly higher than those in matched healthy controls. Testosterone levels were significantly lower than control values for both men (1.98 ± 1.79 vs. 5.28 ± 1.82 ng/ml; *p* < 0.001) and women (0.19 ± 0.15 vs. 0.26 ± 0.11 ng/ml; *p* = 0.008). Similar trends were noted for progesterone (both *p* < 0.001). No significant difference was found in LH levels for both men and women between patients with sTBI and healthy controls.

**Table 2:** Serum hormones.

**Parameters**	**Male patients**	**Male controls**	***P*-value**	**Female patients**	**Female controls**	***P*-value**
Age (years)	35 (23–45)	38 (30–47)	0.182	42 (27–48)	36 (28–45)	0.628
Estradiol (pg/ml)	26.14 ± 18.89	29.59 ± 10.93	0.155	58.50 ± 43.79	94.69 ± 73.66	0.013^*^
FSH (mIU/L)	3.56 ± 3.50	5.27 ± 2.89	0.002^*^	17.76 ± 12.78	8.87 ± 5.93	<0.001^**^
LH (mIU/L)	4.26 ± 2.74	4.76 ± 1.64	0.22	9.79 ± 5.85	9.35 ± 7.52	0.776
Progesterone(ng/ml)	0.25 ± 0.19	0.55 ± 0.21	<0.001^**^	1.20 ± 1.13	4.79 ± 3.84	<0.001^**^
Prolactin(ng/ml)	26.91 ± 14.35	10.00 ± 4.69	<0.001^**^	52.77 ± 23.26	18.89 ± 10.26	<0.001^**^
Testosterone(ng/ml)	1.98 ± 1.79	5.28 ± 1.82	<0.001^**^	0.19 ± 0.15	0.26 ± 0.11	0.008^*^

*FSH, follicular stimulating hormone; LH, luteinizing hormone. Results are given as mean± standard deviation or median (IQR)*.

* *p < 0.05*.

** *p < 0.001*.

### Recovery of Consciousness and Associated Hormone Levels by Sex After sTBI

Of the 92 male patients with sTBI, consciousness was regained in 55 (59.78%) patients. Among these patients, the duration of recovery to consciousness after sTBI was <1 month for 32 patients, 1–3 months for 15 patients, 3–6 months for 6 patients, and more than 6 months for 2 patients. Of the 32 female patients with sTBI, 22 (68.75%) patients had regained consciousness. The recovery to consciousness duration after sTBI was <1 month for 13 patients, 1–3 months for 5 patients, and 3–6 months for 4 patients. There is no statistically significant difference in percentage of the patients regaining consciousness between male and female groups.

Table 3 summarizes the results of single-factor analysis of variables for the RC and NRC groups by sex. There were no statistically significant differences between the two groups in terms of age, GCS, and ISS at baseline for both men and women with sTBI, yet there were statistically significant differences for serum levels of estradiol, FSH, progesterone, prolactin and testosterone. For male patients with sTBI, the RC group had higher levels of FSH (3.40 ± 2.26 vs. 2.13 ± 1.62 mIU/L; *p* = 0.007), higher levels of testosterone (2.69 ± 1.95 vs. 0.93 ± 0.72 ng/ml; p < 0.001), lower levels of progesterone (0.22 ± 0.17 vs. 0.31 ± 0.22 ng/ml; *p* = 0.029), and lower levels of prolactin (23.90 ±11.22 vs. 31.44 ± 17.27 ng/ml; *p* = 0.014) than those for the NRC group. For the female patients with sTBI, the levels of estradiol (76.79 ± 41.12 vs. 18.26 ± 6.50 pg/ml; *p* < 0.001), progesterone (1.66 ±1.09 vs. 0.20 ± 0.07 ng/ml; *p* < 0.001), and prolactin (61.63 ± 22.26 vs. 33.28 ± 9.68 ng/ml; *p* < 0.001) were significantly higher in the RC group compared with the NRC group.

**Table 3:** Single factors analysis of variables between two groups by sex.

	**Male patients**			**Female patients**		
**Variables**	**RC (*n* = 55)**	**NRC (*n* = 37)**	***P*-value**	**RC (*n* = 22)**	**NRC (*n* = 10)**	***P*-value**
Age (years)	33 (23–45)	35 (24–48)	0.687	35 (23–47)	46 (36–52)	0.095
ISS, median	38 (32–46)	35 (28–42)	0.258	35 (28–42)	36 (30–53)	0.411
GCS at admission	5 (3–6)	4 (4–6)	0.711	5 (3–6)	6 (3–6)	0.24
Estradiol (pg/ml)	23.50 ± 17.94	30.05 ± 18.83	0.103	76.79 ± 41.12	18.26 ± 6.50	<0.001^**^
FSH (mIU/L)	3.40 ± 2.26	2.13 ± 1.62	0.007^*^	16.00 ± 12.95	21.63 ± 12.12	0.255
LH (mIU/L)	3.54 ± 2.63	4.67 ± 2.75	0.078	9.35 ± 5.37	10.77 ± 6.99	0.532
Progesterone (ng/ml)	0.22 ± 0.17	0.31 ± 0.22	0.029^*^	1.66 ± 1.09	0.20 ± 0.07	<0.001^**^
Prolactin (ng/ml)	23.90 ± 11.22	31.44 ± 17.27	0.014^*^	61.63 ± 22.26	33.28 ± 9.68	0.001^**^
Testosterone (ng/ml)	2.69 ± 1.95	0.93 ± 0.72	<0.001^**^	0.20 ± 0.14	0.16 ± 0.16	0.436

*GCS, Glasgow Coma Scale; ISS, Injury Severity Score; FSH, follicular stimulating hormone; LH, luteinizing hormone. Results are given as mean± standard deviation or median (IQR)*.

* *p < 0.05*.

** *p < 0.001*.

### Outcome Predictors

We then attempted to evaluate the use of these hormone levels, i.e., the significant differences between the RC and NRC groups, to predict recovery of consciousness by sex, as shown in Table 4. A logistic regression model with recovery of consciousness/no recovery of consciousness as the dependent factor for male patients, which was a combination of the clinical predictors with FSH, progesterone, prolactin, and testosterone as independent factors, showed that testosterone significantly predicted consciousness recovery (OR, 3.495, 95% CI, 1.792–6.815, *p* < 0.001). For the women patients with sTBI, however, sex hormone levels did not contribute to the prediction of consciousness recovery when examining these hormones together with the clinical predictors.

**Table 4:** Logistic regression analysis of variables to predict recovery of consciousness.

	**Multivariate analysis**		
**Variables**	**OR**	**95%CI**	***P*-value**
**MALE PATIENTS**			
FSH (mIU/L)	0.801	0.605–1.060	0.120
Progesterone (ng/ml)	0.367	0.039–3.452	0.379
Prolactin (ng/ml)	0.992	0.980–0.942	0.329
Testosterone (ng/ml)	3.495	1.792–6.815	<0.001^**^
**FEMALE PATIENTS**			
Estradiol (pg/ml)	1.204	0.929–1.559	0.161
Progesterone (ng/ml)	8.944	0.001–200.015	0.341
Prolactin (ng/ml)	0.986	0.953–1.021	0.428

*FSH, follicular stimulating hormone*.

** *p < 0.001*.

**Figure 1:**
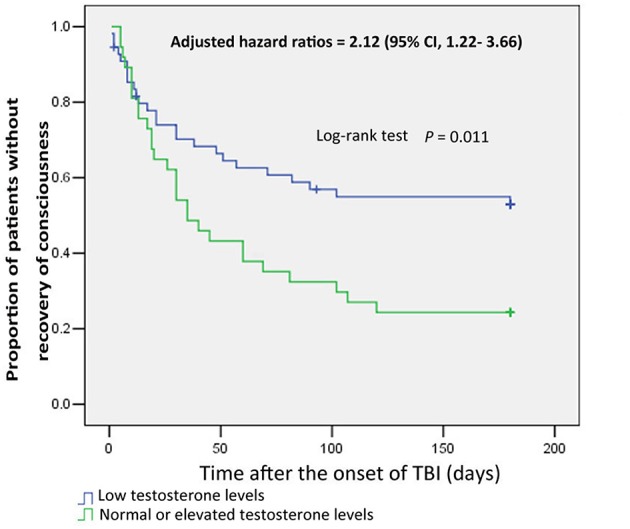
Kaplan-Meier curves of consciousness recovery in male patients with severe TBI, based on testosterone subgroup. Blue curves show the proportion of consciousness recovery in male patients with low testosterone levels. Green curves show the proportion of consciousness recovery in male patients with normal or elevated testosterone levels.

Furthermore, the times to recovery from coma for male patients with normal or elevated testosterone levels and those with low testosterone levels were compared by Kaplan–Meier survival curves, which showed the proportion of male patients regaining consciousness (Figure 1). The analysis showed that normal or elevated testosterone levels in serum significantly reduced the risk of remaining in an unconscious state in male patients with sTBI (log-rank test, *p* = 0.011) (HR, 2.12; 95% CI, 1.22–3.66, *p* = 0.007).

**Figure 2:**
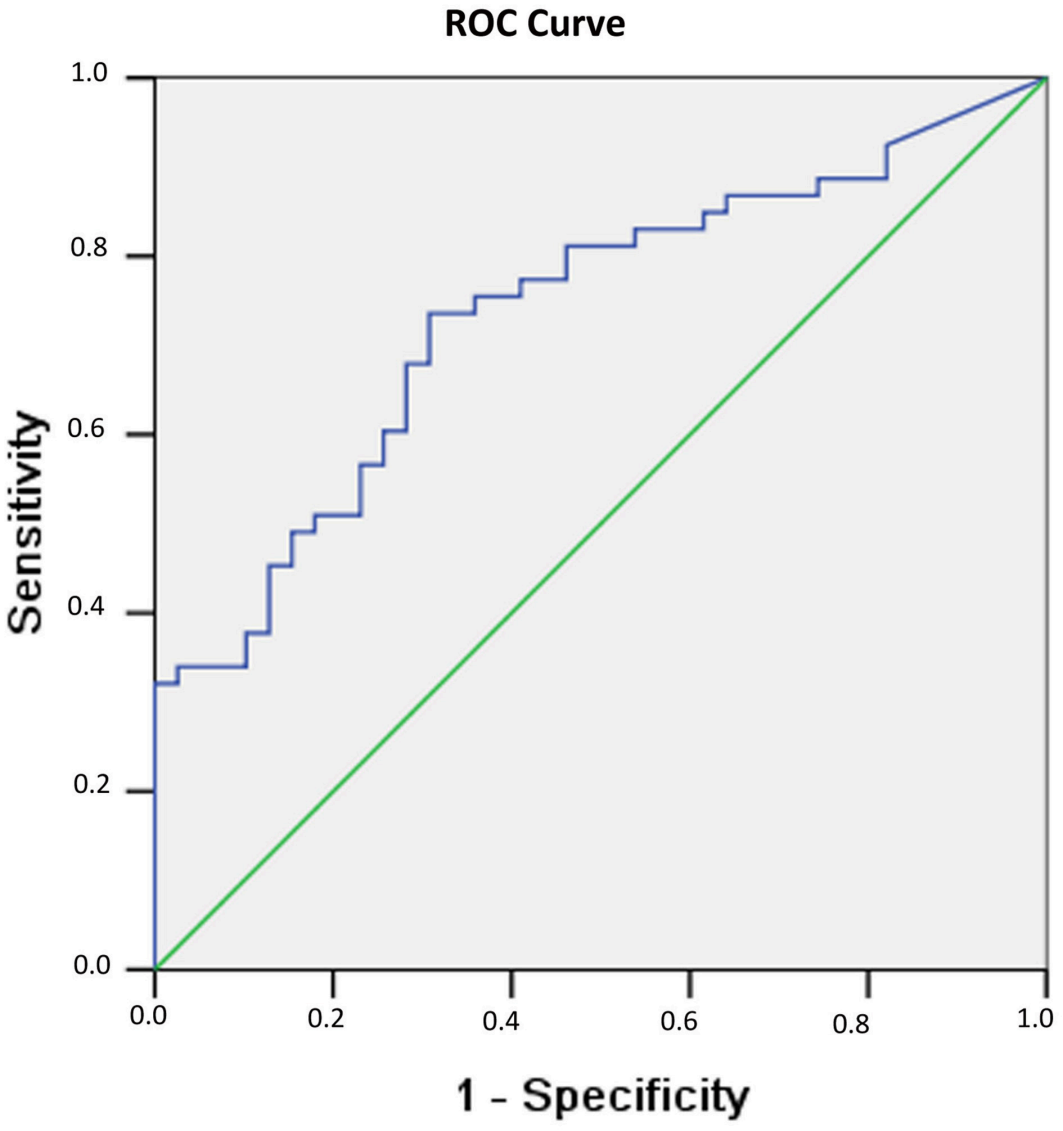
Probability of recovery of consciousness at 6 months related to serum testosterone levels observed 1 week after sTBI in male patients. The probability results are from the ROC curve, where larger test results indicate a more positive test. The AUC for testosterone is 0.736 (*p* < 0.001).

In addition, in evaluating the power of testosterones to predict recovery of consciousness/no recovery of consciousness in men, an ROC curve was drawn, as shown in Figure 2. The ROC analysis showed that the area under the curve (AUC) was 0.736 (*p* < 0.001).

## Discussion

The literature suggests that pituitary hormone abnormalities occur early, with high frequency post-TBI (7, 8). However, these research findings are mostly mixed regardless of sex. Our study investigated alterations in sex hormones and specifically focused on the effects of these hormones on consciousness after sTBI by sex, which has not been well-studied. In the current study, sex-specific alterations in serum sex hormone levels were identified in the acute phase of sTBI. Importantly, our data suggested that serum testosterone was a significant predictor of consciousness recovery in male patients with sTBI, whereas serum sex hormones did not contribute to consciousness recovery in women patients with sTBI.

Hypopituitarism is highly prevalent during the acute phase of TBI. Thus, far, the exact mechanisms underlying hypopituitarism have not yet been clarified. The most widely accepted theory belongs to the ischemic insult to the pituitary gland. Raised intracranial pressure and edema around the region of hypothalamic–pituitary may also contribute to hormonal abnormalities (13). Therefore, it is conceivable that surgical treatment during the acute phase of TBI, such as decompressive surgery operations, could alleviate hormonal abnormalities by reducing intracranial pressure. There is increasing proof that hypopituitarism may be badly neglected in patients with TBI because the lack of routine follow-up of hormone levels (14). In addition, the majority of clinical researches on pituitary abnormalities in TBI to date have been on men because men have a higher incidence of TBI than women or regardless of sex (15, 16). The results of this study further extended previous work examining hormone profiles after sTBI by sex. Our results showed statistically significant changes in FSH, progesterone, prolactin, and testosterone for men patients, whereas in women patients, the changes were observed in estradiol, FSH, progesterone, prolactin, and testosterone. Interestingly, the trend of changes in FSH and prolactin was opposite for sex groups. These findings indicated that TBI differentially affects the levels of sex-steroid hormones in men and women with sTBI. It is frequent to observe sex differences in post-TBI outcomes (17–23). Results from experimental models show that female rats exhibit lesser susceptibility to post-TBI and male rats developed more severe cerebral edema, which could significantly cause secondary brain injury (11). Data from clinical study have noted that women are more likely to survive their injuries and less likely to suffer posttraumatic complications than men (17–20). However, other researchers have found the opposite results that women have worse outcomes and are more likely to die from their injuries than men (18). Sex differences in the extent of brain damage has also been reported among survivors post-TBI, with the female brain suffering from less damage compared to their male counterparts (24). These studies support that pathophysiologic variables may underlie these differences. Numerous studies from clinical and laboratory research support the essential role of sex hormones in the injured brain (20–23). Hence, sex-specific changes of hormonal steroids may contribute to innate sex-based differences in physiology and pathobiology of TBI.

Unconsciousness resulting from TBI is frustrating for clinicians and distressing for patients' families, since the mechanisms behind the recovery from unconsciousness are largely unknown and its prognosis is especially challenging (17). Consciousness is considered to exhibit an emergent property of cortical activity (25). The ascending reticular activating system (ARAS) of the brain structures accounts for the regulation of consciousness (24). It has been proposed that impaired consciousness level post-TBI may be due to damage of part of the ARAS, including the brainstem, thalamus, extensive injury to the cortex, or the disconnection of white matter between the thalamus and cerebral cortex (26). In addition, the hypothalamus plays an important role in maintaining self-awareness since it is involved in the regulation of sleep and awakening as the primary timekeeper of consciousness (27, 28). Hypothalamus-pituitary dysfunction resulting from TBI is mainly caused by damage to the hypothalamus, including hypoxic insult, direct mechanical injury, and vascular injury (7, 14). Hence, we speculated that hormone alterations after sTBI may have a certain degree of predictive value for recovery of responsiveness by combining the above-mentioned studies.

In the current study, we determined how serum sex hormones may be useful for predicting the outcome of unconsciousness. Our results showed that testosterone, only in male patients, was an effective predictor of recovery of consciousness. Notably, normal or elevated testosterone levels were significantly associated with a reduced risk of unconsciousness. Despite the exact mechanism of how testosterone promotes the recovery of consciousness being unknown, several previous studies could support the results from this study. It has been reported that the descent of testosterone is dependent on the severity of TBI. In males, there is a positive correlation between plasma testosterone level and GCS score (29, 30). Moreover, it has also been reported that testosterone level is associated with mortality or morbidity of patients with sTBI (31). Clinical studies suggest that male TBI patients could benefit from restoring serum testosterone levels (10, 32). Beneficial effects of testosterone after brain injury have also been reported in animal experiments. Results of experiment conducted by Lopez-Rodriguez and coworkers show that testosterone levels on brain inversely correlate with the severity of TBI and edema formation, but positively correlate with GCS scores. They also suggest that animals with lower levels of testosterone on brain had higher neurological deficiency (33). Furthermore, brain testosterone plays a neuroprotective effect against oxidative damage in experimental model (34). Other research suggests that intrinsic androgen may impact the capacity of neural stem/progenitor cells to produce neural progenitors under oxidative stress conditions (35). There is evidence that steroid hormones may modulate adult subventricular zone neurogenesis by affecting synthesis of brain-derived neurotrophic factors (36). It has also been reported that testosterone could improve working memory in aged rats by aiding transport of nerve growth factor from hippocampus to cortex (37). Therefore, it is not surprising that testosterone level has an effective predictive value in terms of consciousness recovery. Though male RC group had a significantly higher levels of testosterone and FSH and lower levels of prolactin and progesterone, FSH, prolactin and progesterone were not included in the logistic regression equation. As with females, none of sex hormone was associated with consciousness although RC group presented with higher levels of estradiol, prolactin and progesterone, which may be explained by sex-specific responses to sex hormone. It has also been previously demonstrated that loss of testosterone in men could change the brain's hormonal landscape because alteration of testosterone is gradual in healthy men and can be clinically subtle, whereas change in sex hormones in healthy women is rapid and overt (38). There are notable sex differences in neurochemistry, brain morphology and functional outcomes in addition to similarities between female and male brains (39). Marked sex-specific responses to injury caused by trauma have also been reported in the nervous systems above-mentioned (16–19). These studies may provide evidence for the difference in the association between sex hormones and consciousness for male and female patients post-TBI.

In addition to use of testosterone to distinguish whether patients were likely to have RC vs. NRC, it was of interest to analyze the probability of testosterone levels predicting recovery of consciousness. In the current study, by using testosterone levels in male patients with sTBI the ROC analysis showed a high AUC. The probability of consciousness recovery increased with increasing levels of testosterone, which provides a rationale for why male TBI patients could benefit from restoring their serum testosterone levels as previously suggested in a clinical study (32). There is an increasing belief that unconsciousness following TBI may be the consequence of traumatic axonal injury to the brainstem reticular activating system and thalamus, extensive damage to the cortex (2, 40). Androgens were shown to be an important promoting factor in axons regeneration in males (41). The potential mechanism by which testosterone could enhance consciousness recovery post-TBI was presumably due to facilitating axonal regeneration. However, systemic administration of testosterone to female animal elicited a less extent of axonal regeneration, which could have been due to conversion of testosterone to estradiol by aromatase and subsequently inability to bind to androgen receptors within neurons (42). In addition, effects of sex hormones on brain and behavior can be moderated by factors such as menopausal status, age, and parity (38, 43). These results could contribute to the absence of associations between gonadal hormones and TBI outcomes in women.

In the present study, progesterone and estrogen were not associated with consciousness, although progesterone was significantly lower in both sexes relative to controls, and estrogen was lower only in the female patients compared with health subjects. Decades of researches demonstrate that progesterone can suppress neuroinflammation and reduce edema, oxidative injury, blood-brain barrier damage, enhance dendritic arborization and synaptogenesis, and limit cellular necrosis after brain trauma (8, 44, 45). Experimental literature also suggests that estrogen can increase cerebral blood flow, reduce inflammatory, prevent lipid peroxidation, and promote cell survival post-TBI (11, 46, 47). Despite a growing body of evidence from laboratory studies supporting the influential role of progesterone and estrogen in TBI, there is an alarming paucity of clinical data. The large clinical trials show no clinical benefit of progesterone and estrogen in patients with severe TBI (48, 49). There are currently no recommendation for the use of treatment with estrogen or progesterone to afford neuroprotection in TBI (8, 48). These results may explain that progesterone and estrogen were not associated with consciousness in both sex at current study.

This study was limited by the fact that sex hormone levels were assessed only once and were not evaluated for their dynamic changes. Our data do not allow discrimination between what proportion of the hormone alteration is caused by the TBI itself and how much is caused by the extracranial injuries and critical illness situation. Additionally, the observed indicators were also limited. Functional magnetic resonance imaging and electroencephalography responses, known to provide useful prognostic information, were not included in this study. However, this study was mainly designed to find a biomarker for predicting consciousness recovery at an early stage post-TBI. Our sample size was also relatively small. In the future, more study subjects are needed to overcome possible bias and to improve the generalizability of data.

## Conclusion

The results of this work indicate that acute serum sex hormone profiles are different between male and female patients in the acute phase of sTBI. Serum testosterone concentration is an effective prognostic indicator in male patients with sTBI for recovery of consciousness. Hence, these patients should be considered and referred to neuroendocrine evaluation in an early phase after traumatic event. However, progesterone and estrogen are not significantly associated with the outcome of unconsciousness, so early treatment with progesterone and estrogen may not work on the recovery of consciousness. Further work is needed to investigate the exact mechanism of how testosterone promotes the recovery of consciousness in male population with TBI.

## Ethics Statement

This study was carried out in accordance with the recommendations of Nanfang Hospital, Southern Medical University. The protocol was approved by our Institutional Review Board. All patient's data were analyzed and reported anonymously.

## Author Contributions

YZ and JF designed the protocol. HW and BZ recruited subjects and collected data. RH analyzed the data. YZ drafted the manuscript. JF reviewed and edited the manuscript.

### Conflict of Interest Statement

The authors declare that the research was conducted in the absence of any commercial or financial relationships that could be construed as a potential conflict of interest.
